# The Equivocal Role of Th17 Cells and Neutrophils on Immunopathogenesis of Leishmaniasis

**DOI:** 10.3389/fimmu.2017.01437

**Published:** 2017-10-30

**Authors:** Suênia da C. Gonçalves-de-Albuquerque, Rômulo Pessoa-e-Silva, Lays A. M. Trajano-Silva, Tayná Correia de Goes, Rayana C. S. de Morais, Cíntia N. da C. Oliveira, Virgínia M. B. de Lorena, Milena de Paiva-Cavalcanti

**Affiliations:** ^1^Department of Microbiology, Aggeu Magalhães Research Center, Oswaldo Cruz Foundation, Recife, Pernambuco, Brazil

**Keywords:** cutaneous leishmaniasis, visceral leishmaniasis, immunity, T helper 17, interleukin-17, neutrophil, immunopathogenesis

## Abstract

Advances in the understanding of leishmaniasis progression indicate that cellular interactions more complex than the Th1/Th2 paradigm define the course of infection. Th17 cells are a crucial modulator of adaptive immunity against *Leishmania* parasites acting mainly on neutrophil recruitment and playing a dual role at the site of infection. This review describes the roles of both these cell types in linking innate defense responses to the establishment of specific immunity. We focus on the Th17–neutrophil interaction as a crucial component of anti-*Leishmania* immunity, and the clinical evolution of cutaneous or visceral leishmaniasis. To date, information obtained through experimental models and patient evaluations suggests that the influence of the presence of interleukin (IL)-17 (the main cytokine produced by Th17 cells) and neutrophils during *Leishmania* infections is strictly dependent on the tissue (skin or liver/spleen) and parasite species. Also, the time at which neutrophils are recruited, and the persistence of IL-17 in the infection microenvironment, may also be significant. A clearer understanding of these interactions will enable better measurement of the influence of IL-17 and its regulators, and contribute to the identification of disease/resistance biomarkers.

## Introduction

*Leishmania* parasites are transmitted to mammals by female phlebotomine sandflies and cause a group of diseases with symptoms defined mainly by the parasite species and the host’s ability to develop and control immune responses (1). Sickness results from uncontrolled infection, and the wide spectrum of clinical manifestations (from healing cutaneous lesions to fatal visceral infections) is associated with the parasite species involved, among other factors (2). The main clinical forms are cutaneous (e.g., *Leishmania major, Leishmania tropica, Leishmania mexicana*, and *Leishmania braziliensis*), mucocutaneous (e.g., *L. braziliensis*), and visceral (*Leishmania donovani* and *Leishmania infantum*) leishmaniasis (3). Visceral leishmaniasis (VL) is the most severe form and may compromise important organs and tissues, such as the liver, spleen, bone marrow, and lymph nodes, leading to hepatosplenomegaly, lymphadenopathy, anemia, constant fever, and immunosuppression (4). Cutaneous leishmaniasis (CL) is characterized in most cases by well-delimited ulcerated skin lesions with raised borders (5). Mucocutaneous leishmaniasis (ML) usually compromises mucosal regions of the nose, mouth, and pharynx, and is associated with disfiguring and psychosocial sequelae (6, 7). There are estimated to be 12 million people currently living with leishmaniasis, and the World Health Organization estimates that there are approximately 0.7–1.2 million new cases of CL and 0.2–0.4 million cases of VL per year (6). While CL and ML are associated with social stigma, VL may result in fatality rates of 10–20% if not treated (6).

The parasites have a digenetic lifecycle and two distinct morphologies: the promastigote in the sandfly vector midgut, and the amastigote in mammalian host phagocytes. The motile, flagellated promastigotes exist, multiply, and develop extracellularly in the alimentary tract of blood-sucking female sandflies and are transmitted into the mammalian host during blood meals. Inside the host, they infect macrophages in the reticuloendothelial tissue and differentiate into non-motile amastigotes, and multiply as such in phagolysosomal vacuoles (8).

The interaction of inflammatory and regulatory responses delimited by cell-mediated immune responses drives disease expression and may result in asymptomatic infection, self-healing, or chronic leishmaniasis (1). Pathogenesis follows a complex set of interactions between factors triggered by the host’s innate and acquired immune responses, which are strongly influenced by some aspects such as the host’s genetic background (9, 10), infecting species, dose and route of inoculation (1), and sandfly saliva components (8). Only 2–3% of individuals infected with parasites of the *Viannia* subgenus (*L. braziliensis, Leishmania guyanensis, Leishmania panamensis*, and *Leishmania peruviana*) develop skin lesions (11) and less than 10% of those infected with *L. donovani* or *L. infantum* develop active VL (12). The relationship established between *Leishmania* parasites and the vertebrate host can lead to a self-healing infection or to the clinical manifestations of leishmaniasis with different severity grades (4). The inflammatory responses mediate disease presentation and, in both forms of the disease, the different clinical manifestations are a function of parasite replication and the efficacy of the immune response generated (5). Clinical cure is associated with the activation of macrophages into a leishmanicidal state mediated by some specific proinflammatory cytokines (1). However, there is as yet no consensus on the mechanisms that lead to susceptibility in humans.

Cellular immunity generated by the T helper type 1 profile is considered to be the key mediator of resistance to *Leishmania*. A protective immune response against CL caused by *L. major, L. mexicana*, or *Leishmania amazonensis*, as well as VL caused by *L. donovani* or *L. infantum*, depends on the development of the proinflammatory T cell profile (13). However, exacerbated Th1 responses may cause severe tissue damage and are also responsible for the clinical presentation of the disease. High levels of Th1 cytokines in people with localized CL lesions are associated with disease outcome, because individuals infected with *L. braziliensis*, who do not develop the disease, produce less interferon (IFN) γ and tumor necrosis factor (TNF) (14). *L. braziliensis* infections are characterized by excessive production of proinflammatory cytokines such as IFNγ, TNF and interleukin (IL)-6 (12) and have lower levels of Foxp3^+^ cells (regulatory lineages) and IL-10 than those infected by other *Leishmania* species (15). In fact, the absence of IL-10-mediated regulation of Th1 is more significant in disease development due to *L. braziliensis* infection than the Th2 polarization itself (16). Indeed, high levels of IL-4 are not observed in patients with severe diffuse CL, suggesting Th2 responses may have less influence on disease progression in humans than in animal models (17).

Interleukin-10 is an important regulatory cytokine that inhibits phagocytosis and affects the ability of macrophages to kill intracellular parasites, contributing to the growth and spread of *Leishmania* (18). IL-10 sources have been identified as CD4^+^/CD25^+^ T cells (Th2) (19, 20), CD4^+^/CD25^−^/FoxP3^+^ regulatory T cells (Tregs) (21), and CD4^+^/CD25^−^/FoxP3^−^ T cells (Th1) (16), stimulated by the recognition of amastigote antibodies (22). Together with other cytokines from these regulatory populations, IL-10 plays a central role in promoting an alternative kind of macrophage activation, which increases arginase expression and facilitates parasite expansion (13).

A successful cellular immune response, which allows parasite elimination without tissue damage, requires a balance between the set of cytokines secreted in the cellular response, beginning with the recognition of the parasite by antigen-presenting cells (APCs) and antigen presentation, the production of Th1 differentiation cytokines, the activation of IFNγ/TNF-producing Th1 cells, and classical macrophage activation (23), ending with the activation of Th2 cells, which mainly secrete IL-10 and block nitric oxide (NO) production by macrophages (13). However, even in the presence of effector molecules such as IFNγ, TNF, and NO during active disease in humans, parasite multiplication persists in many cases. Ansari et al. (24) suggested that despite high IFNγ levels during *L. donovani* infection, the host fails to control the disease due to an incomplete response to IFNγ. Similarly, high titers of TNF have been observed in sera from patients with active VL and diffuse CL, characterized by high parasite loads (25). The biological network acting for equilibrium between parasite clearance and tissue preservation in humans involves substantial participation from effector T cells other than Th1 and Th2 (26).

Helper T cell responses are now known to include four T cell subsets: Th1, Th2, Th17, and Tregs. Th17 cells have recently emerged as an independent T cell subset that may play an essential role in protecting against certain extracellular pathogens (27). However, this relatively newly discovered (28) T cell population has been demonstrated to influence the balance between inflammatory and anti-inflammatory cytokines, which must be orchestrated in the course of infection to guide a successful effector response to the intracellular *Leishmania* protozoa (13). Particular interest in the Th17 type remains focused on its main cytokine, IL-17. Although it is also produced by other cells including CD8^+^ T cells and neutrophils, IL-17 is mostly produced by Th17 cells. IL-17 is part of a complex mechanistic web that involves up- and downregulation of anti- and proinflammatory cytokines as well as interferences in the genetic background of the host. Furthermore, IL-17 performs an important function in neutrophil recruitment (29). Since neutrophils are regarded as an important element during *Leishmania* infection (30–33), it would be reasonable to consider that Th17 cells might have a significant role in the complex diseases caused by these parasites. The crosstalk between the neutrophils and APCs present at the site of infection contributes to the type and magnitude of the specific immune response that will develop (30, 34). Nevertheless, at the site of infection, the protecting or damaging role of the neutrophil depends on the parasite species, host, and phase of infection (35). Thus, the effects of Th17/IL-17 are still unclear, as disease-promoting and protective responses have both been attributed to their influence. In this scenario, the Th17 cell activities in the context of the different clinical forms of leishmaniasis, interaction with regulatory cytokines, as well as host particularities, remain to be explored (13, 36).

Although many excellent reviews have discussed the cellular immune responses elicited against the different forms of leishmaniasis (35, 37, 38) or the roles of Th17/IL-17 in infectious and non-infectious diseases (39–42), reviews of the participation of IL-17 and Th17 participation in immunopathogenesis and control of the main clinical forms of leishmaniasis remain scarce (36). In the present review, we identify the Th17–neutrophil interaction as a crucial component of anti-*Leishmania* immunity. A better understanding of immune complexity will contribute to the identification of disease/resistance biomarkers and influence the development of vaccines and immunotherapies for leishmaniasis.

## Th17 Cells and IL-17

The recent recognition of Th17 cells has provided new insights into the mechanisms that are important in autoimmune diseases and antimicrobial host defenses (28, 43). Th17 cells represent a subset of CD4 effector T cells distinct from Th1 and Th2 lineages, and mediate powerful effects on stromal cells; this results in the production of inflammatory cytokines and recruitment of leukocytes, especially neutrophils, creating a link between innate and adaptive immunity (29). *In vivo* studies indicate that IL-17 is an especially potent activator of neutrophils, both through expansion of the lineage and through their recruitment *via* chemokine expression regulation (44). IL-17 cooperates with other cytokines secreted by Th17 (such as IL-17F,^1^ IL-21, and IL-22) to induce tissue inflammation, leading to different effector functions depending on the pathogen (47).

**Figure 1:**
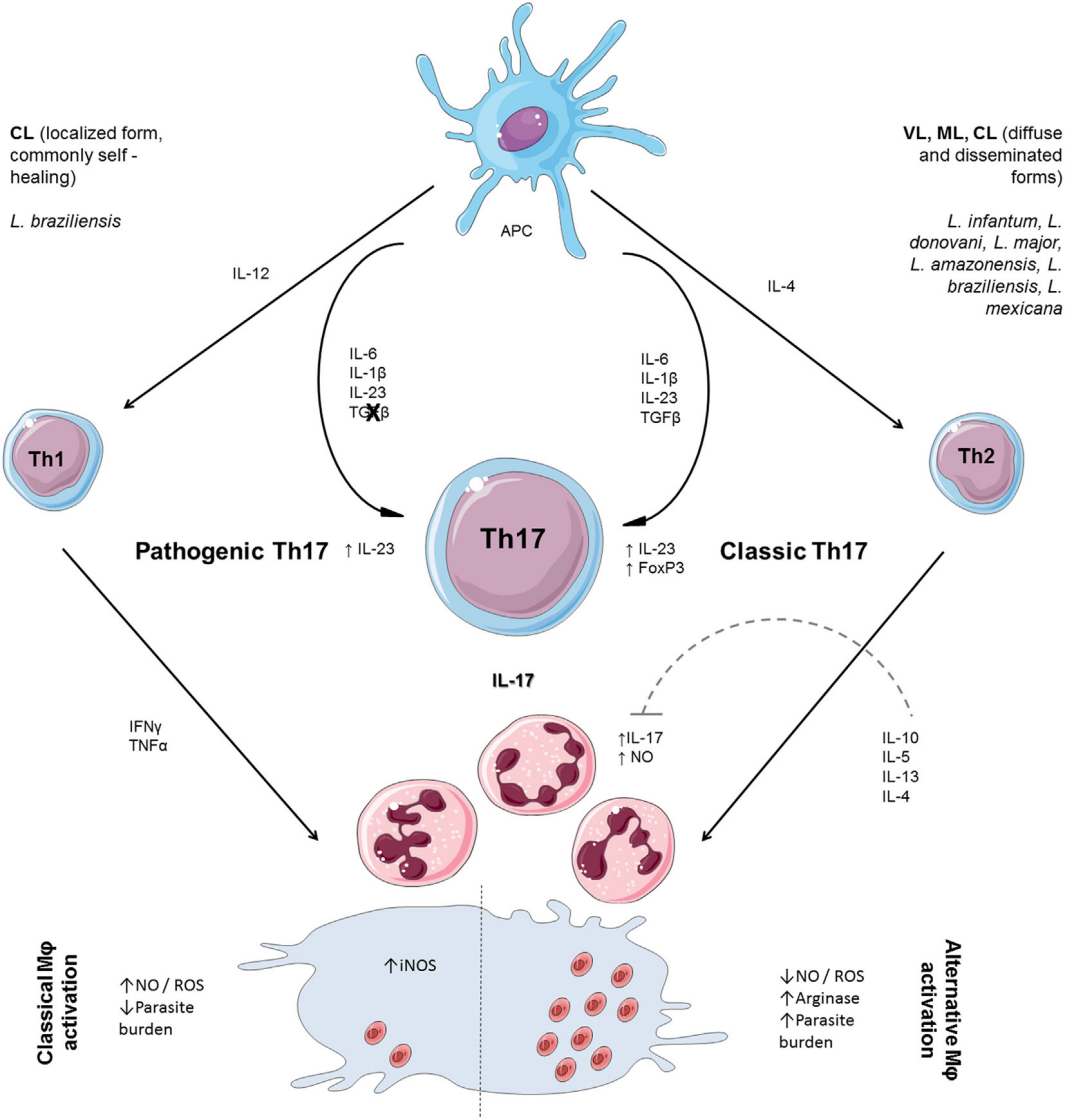
T helper cell polarization and participation of neutrophils on effector responses to *Leishmania*. Th17 cell differentiation is mediated by activation of naïve T cells in a combination of TGF-β, interleukin (IL)-6, IL-1β, and IL-23. TGF-β and IL-21 upregulate the IL-23 and IL-23 receptor, decisive for the maintenance of Th17 cells. Th17 cells can also be generated independently of TGF-β, in an alternative manner. When “classical” Th17 cells were compared with alternatively induced Th17 cells, the “alternative” Th17 cells were more pathogenic. Naïve CD4^+^ T cells differentiate to the Th1-cell lineage in the presence of IL-12. Interferon (IFN) γ secreted by Th1 activates inducible NO synthase (iNOS) and enhances production of nitric oxide (NO) and reactive oxygen species (ROS), required for parasite clearance. The production of ROS in the infection microenvironment is enhanced by the presence of neutrophils recruited by IL-17 and, in the absence of IL-10 regulation, may worsen tissue injury. The secretion of IL-4 leads to transcription of Th2 genes (IL-10 and IL-13) broadly associated with susceptibility to *Leishmania* infection through alternative macrophage activation, favoring parasite persistence. IL-10 is an important regulatory cytokine that controls Th17 differentiation and regulates NO production. The influx of neutrophils plays a positive role by participating in parasite killing and is limited by the presence of IL-10.

Th17 cell differentiation in both humans and mice is mediated by the activation of naïve T cells in the presence of a combination of TGF-β, IL-6, IL-1β, and IL-23 (48, 49). IL-6 acts in concert with TGF-β to induce the development of Th17 effector cells. Together, TGF-β and IL-21 upregulate the IL-23 and IL-23 receptors, a decisive step in the full differentiation and maintenance of Th17 cells (50). Although the requirement of TGF-β for the classical generation of Th17 cells *in vitro* and *in vivo* has been demonstrated, as well as the endogenous production of TGF-β for autocrine stimulation (51), it has also been shown that Th17 cells can be generated independently of TGF-β (52), in an alternative manner. Thus, the availability of IL-23 and/or TGF-β has emerged as a determinant of Th17 effector phenotypes; in other words, an abundance of TGF-β with a relative lack of IL-23 favors the generation of “classical” Th17 cells, while the presence of IL-23 alone promotes the generation of “alternative” Th17 cells. Transcription factors that are differentially expressed in “classical” vs. “alternative” Th17 cells will cause differential cytokine expression and effector functions. When “classical” Th17 cells were compared with alternatively induced Th17 cells, the “alternative” Th17 cells were more pathogenic (51, 52) (Figure 1).

Th17 differentiation is dependent on the steroid receptor-type nuclear receptor (RORγt) (47), as the transduction of naïve T cells with a retroviral vector containing RORγt induces the development of IL-17-producing T cells (53). The IL-23 receptor activates signal transducer and activator of transcription (STAT) 3, which induces RORγt (54). Conversely, in murine models, the generation of Th17 cells is inhibited by IL-4, IL-10, and IFNγ, possibly *via* downregulation of the IL-23 receptor (54, 55). The reciprocity of this antagonism from Th1/Th2 cells remains poorly understood, but as well as Th1 being counterbalanced by Th2 cells (56), Th17 cells are reciprocally related to Foxp3^+^ Tregs. The presence of TGF-β activates the transcription factor Foxp3 in naïve T cells, whereas the presence of IL-6 suppresses Foxp3, and combined with TGF-β induces RORγt, leading to Th17 differentiation. Thus, the balance between Th17 cells and Foxp3^+^ Tregs is mediated by the antagonistic interaction of the transcription factors Foxp3 and RORγt [reviewed by Korn et al. (47)].

Th17 is primarily known for its enhancement of host protection against extracellular bacteria and fungi, which requires robust inflammatory infiltrates that are not efficiently cleared by Th1 and Th2 responses (47, 57). IL-17 acts by activating iNOS and inducing the expression of granulocyte macrophage colony-stimulating factor, IL-1β, IL-6, IL-8, TNF, and several chemokines, which collaborate to potentiate the inflammatory reaction (58). The induction of these inflammatory mediators suggests that IL-17 plays a role in intracellular parasite infections. Indeed, the differentiation of Th17 subsets has been noticed during infections of *Mycobacterium tuberculosis* (59), *Francisella tularensis*, and *Listeria monocytogenes* (60). The role of IL-17 is also essential for protection against *Trypanosoma cruzi* in the acute phase of Chagas disease (61, 62), suggesting that Th17 cells are important in the successful clearance of kinestoplastid protozoans such as *Leishmania*. However, although Th17 cells and IL-17 have been shown to protect against some intracellular pathogens including *Leishmania* (63, 64), many recent findings point to them as being responsible for excessive inflammation and pathology (65, 66). Currently available data from VL and CL studies are discussed below.

### IL-17 in VL

Active VL is associated with the upregulation of Th2 cytokines and disease progression. Neutrophil depletion during experimental VL results in enhanced parasite loads (67). The effector role of IL-17A is mediated by the accumulation of neutrophils, the enhancement of IFNγ secretion, and the increased production of antimicrobial peptides, acute phase proteins, mucins, and matrix metalloproteases (45). As IL-17 is associated with neutrophilic inflammation and enhancement of the proinflammatory response, the positive role of this cytokine in VL has been investigated. It is already known that *L. donovani* and *L. infantum* stimulate the differentiation of Th17 cells in peripheral blood mononuclear cells from healthy subjects, as well as the production of IL-6 and IL-23 required for Th17 maintenance (68). A cohort study of individuals with VL showed that IL-17 is strongly associated with protection. This protective role is associated with the increase of CXCL chemokines, which serve as potent chemoattractants for neutrophils and Th1 cells. Thus, naturally resistant subjects, showing greater IL-17 responses, would react more rapidly to *L. donovani*, not only by attracting innate immunity responses, but also by recruiting Th1 cells to tissues (68). In BALB/c mouse models of *L. donovani* infection, parasite clearance was associated with the secretion of Th1 cytokines along with IL-17 and IL-23. The blocking of stimuli by anti-IL-17 and anti-IL-23 led to increased parasite loads in the liver and spleen. Subsequent administration of recombinant IL-17 generated higher levels of IFNγ and NO than recombinant IL-23 did, leading to a stronger association of IL-17 with parasite clearance (69). These data corroborated previous findings (68) that Th17 potentiates the Th1 response. Depending on the infectant parasite load, the speed of Th1 recruitment and the quantity of NO generation may determine disease progression or natural control.

mRNA analysis revealed greater IL-17A production in VL patients than in healthy controls, in a study performed by Nascimento et al. (55). In the same study, the highest production of IL-17A coincided with the parasitic peak in infected C57BL/6 mice. These data demonstrate the stimulation of IL-17 production by *L. infantum*, promoting the control of parasite replication by strengthening T helper type 1 responses and NO production. The authors also demonstrate that the absence of IL-17 signaling leads to the generation of a Treg/IL-10-dominated response and an impaired Th1 profile, resulting in parasite growth. However, although IL-17 receptor *knockout* mice were more susceptible to infection and also exhibited reduced inflammatory infiltration, the high levels of this cytokine observed in humans was not sufficient to generate IFNγ/NO levels that lead to natural recovery from VL (55). Thus, although the presence of this cytokine has been demonstrated, the significance of its participation in the effector response seems to be relative to the host.

Neutrophil influx mediated by IL-17A was revealed by Quirino et al. to be an important mediator of parasite clearance during VL (4). In this work, the authors indicate that IL-27 is an important regulator of Th1 and Th17 profiles in a VL mouse model. Composed of two subunits (IL-27p28 and Ebi3) that bind to IL-27 receptor, leading to STAT-1 (Th1) and -3 (Th17) activation, IL-27 plays a role in the regulation of the immune response (4). The authors observed that Ebi3^−/−^ mice present a peak in neutrophil migration at 4 weeks post-infection, both in the spleen and in the liver. These data reinforce the previous findings of Lopez Kostka et al. (70) that suggest that the IL-17–neutrophil axis has more of an influence in the recovery from VL infection after its establishment (when the antigens are already recognized and presented by DCs and macrophages) than in the prevention of infection. Neutrophils recruited by DCs in a toll-like receptor 2 (TLR2)-dependent manner are efficient producers of NO and provide a prototypal Th1 and Th17 environment, leading to a decrease in parasite number in C57BL/6 mice infected with *L. infantum*. Furthermore, TLR2^−/−^ mice are unable to recruit neutrophils in this way and had higher levels of IL-10, indicating that a non-responsive neutrophil state is associated with parasite persistence (71). The presence of neutrophil infiltrate, however, may cause damage to the tissue, and seems to be most significant in skin, since the Th17 markers IL-23, IL-17, and RORγt were highly expressed during post kala-azar dermal leishmaniasis (PKDL) compared with controls (72).

The cell source of IL-17A expression is another variant to be studied. IL-17A-knockout C57BL/6 mice are highly resistant to VL infection, showing fewer parasites in the liver and spleen. This phenotype was associated with enhanced IFNγ production by T cells and decreased accumulation of neutrophils and monocytes, resulting in fewer granulomas. The source of IL-17 was mainly γλ T cells, which commonly express markers for CD3, but not CD4 or CD8 (67), and are differentiated by IL-1β and IL-23, suggesting a different mode of activation to the conventional TGF-β and IL-6 that promote CD4^+^IL-17^+^ cells. IL-17 produced by γλ T cells suppresses NO-mediated parasite control in macrophages in the liver within 7 days of infection, contributing to susceptibility in C57BL/6 mice (73). The mechanisms that lead to counter-regulation between IFNγ and IL-17 secreted by γλ T cells remain to be elucidated.

### IL-17 in CL

*Leishmania* infection strongly stimulates IL-17 production. Elevated levels of IL-17 have been measured in patients with CL and ML, showing that Th17 activation is generated by *L. major* (74, 75), *L. braziliensis* (64), *L. tropica* (26), *L. panamensis* (64), *L. guyanensis, L. amazonensis*, and *Leishmania naiffi* (66) infections, acting on macrophage activation and neutrophil recruitment. However, clinical studies indicate differences in immune responses during CL between the subgenera *Viannia* and *Leishmania* (76). For example, higher levels of Th17 were found in the sera of patients infected with *L. (Viannia) guyanensis* than in those infected *L. (Leishmania) amazonensis* (66). Nevertheless, the mechanisms of immune evasion used by *L. amazonensis* are known to block cellular immune responses (37) and may also occur by decreasing Th17 activation. Thus, the influence of Th17/IL-17 on disease depends on the species. As studies on IL-17 are relatively recent, most performed to date have evaluated *L. major* models or *L. braziliensis* infections in patients. Comparing IL-17 participation in other *Leishmania* species seems, therefore, to be an interesting avenue of investigation.

Proinflammatory responses, especially Th1, are a required component in controlling leishmaniasis. However, in the case of CL and ML, the immune response itself contributes to lesion progress. In both these forms, patients develop intense inflammatory responses that damage tissues, despite the low number of parasites in lesions (25), so the disease is mostly associated with a failure of anti-inflammatory cytokine secretion, especially IL-10 (77). IFNγ and IL-17 levels are substantially elevated in mice lacking the capacity to respond to IL-10. According to Gonzalez-Lombana et al. (2), in these mice, IFNγ does not seem to contribute to pathology as much as IL-17, since the former promotes mostly monocyte infiltration, while the latter recruits neutrophils, constituting a nonspecific response. Interestingly, blocking IFNγ increases IL-17 level and pathology, supporting the hypothesis that, in C57BL/6 mice, IFNγ may be critical for downregulating the responses of IL-17 as well as IL-10 (2). In another study, self-healing of the infection in mice correlated with the expansion of IFNγ and IL-17-producing CD4 cells, suggesting the existence of other active mechanisms to regulate local inflammation (7).

In experimental models, lesion progression is related to IL-17-mediated neutrophil recruitment. During the course of infection with *L. major*, BALB/c (a susceptible strain) CD4^+^ cells and neutrophils produced more IL-17 than did cells from (resistant) C57BL/6 mice. IL-17-deficient mice had decreased neutrophil infiltration and smaller lesions than controls (70). Anderson et al. (74) found an association between severe pathology and the presence of IL-17^+^CD4^+^ cells, which was also linked to an increase in cellular infiltrate expressing macrophage and neutrophil markers. However, despite contributing to lesion exacerbation, no beneficial effect of controlling parasite replication was observed. Conversely, neutrophils have been thought to contribute to parasite clearance in the early stages of experimental *Leishmania* infection (30), and their expression is closely, but not uniquely, related to IL-17 secretion (78). Thus, neutrophil participation must be better studied and linked to IL-17 associations with leishmaniasis. In resistant strains of mice, the number of polymorphonuclear leukocytes decreases to 2% within 72 h of infection, when the specific immune response is defined. In contrast, insusceptible BALB/c mice, neutrophils continue being recruited and have been detected in large numbers at the site of infection more than 10 days after parasite inoculation (30). Pedraza-Zamora et al. (79) showed that in BALB/c mice, Th17 cells and neutrophils both increased up to 90 days post-infection with *L. mexicana*. This suggests that chronic inflammation throughout *L. mexicana* infection is a consequence of neutrophil recruitment together with Th17 cell differentiation throughout the late phase of the infection. C57BL/6 mice show a more controlled response of these cells and a more resistant infection profile. Together, these data suggest that not only the level of IL-17 but also its persistence during infection resolution, is a decisive factor in disease progression. The excessive activation and maintenance of Th17 by the presence of neutrophils and Th1 may cause a damaging block of regulatory populations and IL-10 secretion, which is necessary to stop inflammation. For example, the study performed by Lopes Kostka et al. (70) suggested that DC-derived IL-23 maintains IL-17 production, thus being responsible for disease progression.

mRNA expression of RORγt and IL-17 is observed in patients with ML, and the synergy of IL-17 and IFNγ is suggested by a positive correlation between IL-17 and IFNγ mRNA expression in ML lesions caused by *L. major* (75), contrasting with the antagonism observed in C57BL/6 mice (2) mentioned earlier. Enzyme-linked immunosorbent assays showed that IL-17 secretion by peripheral blood mononuclear cells was elevated in patients with CL and ML compared with uninfected individuals, and downmodulated by IL-10 and TGF-β (65, 80) similarly to that seen with Th1/IFNγ, providing further evidence for the existence of IL-17/IFNγ synergy in humans. de Assis Souza et al. (63) showed that levels of IL-17 were significantly higher in patients infected by *L. braziliensis* during active disease than in those who recovered without intervention. Interestingly, the authors associated this decreased expression during healing to a suppressive role exerted by high NO levels. During treatment, NO levels decrease and allow more IL-17 secretion (63). In addition, the number of IL-17^+^CD4^+^ cells at lesion sites in patients with CL and ML directly correlates with the number of inflammatory cells, supporting their participation in neutrophil infiltration at lesion sites (65).

Although these important studies have linked the presence of IL-17 to more severe clinical presentations of disease, neutrophils are the first line of defense and are also capable of producing NO and killing parasites (32). Therefore, the presence of neutrophils in this inflammatory context can indicate either protection or injury. The reversal of immunodeficiency in *L. panamensis-*infected mice leads to an increase in IFNγ and IL-17, allowing parasite control and elimination (64). This suggests that in the absence of anti-inflammatory cytokines (such as IL-10, IL-13, and IL-4), Th1/Th17 cells proliferate simultaneously and are responsible for parasite elimination, although neutrophil migration, and its role in this situation (3.5 weeks post-infection), was not assessed. This observation reinforces the differences in immunomodulation between parasite species. Interestingly, Novoa et al. (81) also found a trend toward increased levels of IL-17 in human subclinical *L. braziliensis* infections compared with symptomatic CL, suggesting no pathological role of IL-17 in CL or ML. Although those authors associated spontaneous cure to a weak type 1 immune response (due to quick parasite control by innate immune responses), IL-17 levels were measured in the study only 96 h after soluble antigen stimuli, when the cellular immune response was probably being established. The expression of IL-27 (which can act as regulator of IL-17) was also higher in patients with CL caused by *L. braziliensis* than in subclinical individuals (81), reinforcing the idea that the Th17 profile contributed to cure in this study, and supporting a role of IL-17 in parasite depletion. Further investigations encompassing the absence of other IL-17 regulatory mechanisms (such as IL-13) in this situation are necessary.

## Neutrophils in Innate and Adaptive Immunity to *Leishmania*

Neutrophils are cells that operate in innate immunity as the first line of defense, and play an important role in the elimination of pathogens (82). Toxic granule release from the site of infection, secretion of cytokines, phagocytosis, and formation of fibrous structures, known as NETs (neutrophil extracellular traps) are some of the mechanisms used by these cells to eliminate microorganisms (82, 83). In addition to their role in the elimination of pathogens, neutrophils can also participate in immune regulation as a source of cytokines, including IL-2, IL-10, IFNγ, and TNF, thereby establishing a link between innate and adaptive immunity during infection, and even participating in the presentation of antigens to regulate T cell proliferation and antibody production by B cells (84). In fact, murine *L. (L.) amazonensis*-infected macrophages cocultured with neutrophils were better able to destroy the intracellular amastigotes (34). Since no significant difference in *L. (L.) amazonensis* parasite destruction was observed between co-cultures from susceptible BALB/c and resistant C3H/HePas mice, this leishmanicidal activity seemed not to depend on adaptive immunity, which determines an individual’s resistance profile (34). However, due to the effector functions in the inflammatory process, neutrophils also need to be regulated because their excessive activity can lead to tissue destruction and even chronic inflammation (82).

Recent studies have addressed the role of neutrophils in *Leishmania* spp. infection. These cells may behave protectively or as enhancers of infection, depending on the parasite species, host, and stage of infection (32, 34). *In vitro* studies demonstrated that several *Leishmania* species are able to induce the formation of NETs, which are basically DNA structures coated with antimicrobial molecules, released by neutrophils (85). These NETs are involved in the sequestration, immobilization, and killing of parasites [reviewed by Bardoel et al. (86)]. However, the efficacy of this immune strategy varies according to the *Leishmania* species involved. For instance, promastigotes of *L. amazonensis* showed susceptibility to NETs, while *L. donovani, L. infantum*, and *L. mexicana* were able to escape from this “trap” using different mechanisms (82). Furthermore, the same promastigote surface lipophosphoglycan that is involved in the activation of NETs (as for *L. amazonensis*) may also be associated with parasite resistance to killing through NETs, as occurs with *L. donovani* (87).

There is no consensus on the activity of neutrophils during infection by *Leishmania* species, as besides the complexity of species involved, there is heterogeneity between studies, which explore different host species, host strains, and tissue types, all factors that may interfere directly with the type of response to the infection (32). In an example of how the host strain (genetic background) may change its response, Tacchini-Cottier et al. (33) demonstrated that neutrophil-depleted C57BL/6 mice exhibited a normal course of infection caused by *L. major*, whereas neutrophil depletion in BALB/c resulted in blockage of the early characteristic IL-4 response, thus leading to an inhibition of Th2-type response. Currently, a specific neutropenic mouse strain (Genista) is being used; Hurrell et al. (82) demonstrated that Genista mice, as well as mice with transient neutrophil depletion, are able to control the parasite load and lesion size when infected with *L. mexicana*, indicating that parasite sequestration by neutrophils is associated with disease progression in mice.

Neutrophils that are recruited and that participate in the early stages of infection behave very differently to those associated with the late immune response against *Leishmania* parasites. In the initial phase of infection, neutrophils are recruited as a response to chemotactic substances released from the tissue lesion or from the sandfly’s saliva, and also by stimuli from the parasites (32). DCs and neutrophils have been shown by microscopy studies to infiltrate the site of parasite inoculation within a few hours after sandfly bite, where they engulf the parasites. By 48 h after infection, most parasites at the inoculation site are found within macrophages (88). DCs that were recovered from the skin 1 day after infection appeared to have been infected by these parasitized neutrophils (32). *In vitro* and *in vivo* kinetics studies showed that interactions with early infected or not infected neutrophils (from the acute infiltrate), or even with their apoptotic corpses, may impair the innate response of DCs, thus delaying the activation of a specific immune response *via* CD4^+^ T cells (32). This response may be crucial for parasite depletion before symptom presentation, resulting in subclinical progression. The parasites may enter directly into the macrophages through receptor recognition, or may be transferred silently *via* neutrophils (89). Parasites may be transferred from neutrophils to macrophages in the following two ways: the first involves the release of the parasite from an infected apoptotic neutrophil, in which the free parasite is phagocytosed by the macrophage; the second suggests that the macrophage engulfs live infected neutrophils, referred to as “Trojan horses” (90, 91). Mollinedo et al. (92) have demonstrated viable *L. major* and *L. donovani* promastigotes inside human neutrophils, and they suggest that this is linked to inhibition of the oxidative burst, as well as prevention of early fusion of lysosomes with parasite-containing phagosomes. The interaction and previous uptake of these early neutrophils by macrophages can drive the development of IL-10-high and IL-12-low macrophages, which are especially permissive to the survival and proliferation of *Leishmania* parasites (32).

Antigen presentation by neutrophils is also inefficient in eliciting adequate T cell differentiation. Recent work revealed that human leukocyte antigen–antigen D related (HLA-DR) positive neutrophils from subjects with VL did not stimulate T-cell proliferation, but they did show higher expression of programmed cell death ligand-1 than other neutrophils, and lymphocytes of the same subjects showed high expression of programmed cell death protein-1, reinforcing the mechanisms of apoptosis that favor *Leishmania* (93). This population of neutrophils also showed elevated expression of IL-10 mRNA and protein, and transcripts encoding arginase-1, which is involved in the suppression of T cell responses (94). Conversely, after *in vitro* infection with *L. braziliensis*, neutrophils from patients with CL produce more reactive oxygen species (ROS) and higher levels of chemokines associated with recruitment of neutrophils and Th1-type cells than neutrophils from healthy control subjects (95), highlighting that the mechanisms used by parasites to explore neutrophils in their favor vary by species.

After the first acute neutrophilic influx, a second wave of neutrophil recruitment to the site of infection occurs approximately 1 week after the first contact with parasites; this is especially mediated by IL-17. Studies using BALB/c IL-17-deficient mice suggest that this cytokine is not the main recruitment factor involved in early stages of infection, but acts beside IL-8 and granulocyte-colony-stimulating factor. However, after 4 weeks of infection, low levels of IL-17 lead to a low number of neutrophils (70). Thus, the role of IL-17 in both leishmaniasis forms (cutaneous and visceral) may be strictly associated with neutrophil function, infection stage, and antioxidant capacity, as well as IL-10 regulation. When the neutrophil is engulfed by an infected macrophage, parasite death is increased *via* an elevation in ROS production. However, this second response is also associated with lesion progression, especially in the mucosal form of leishmaniasis (2, 30). According to Gonzalez-Lombana et al. (2), the key point in the healing of a lesion in ML is the downregulation of IL-17 by IL-10 and IFNγ. The huge concentration of granules or products from neutrophils recruited by IL-17 in the site of infection promotes tissue lesioning. In this context, the authors suggest that the IL-17 pathway is an important therapeutic target for the treatment of severe leishmaniasis in patients in whom IL-10 regulatory function is compromised.

In experiments performed in BALB/c mice, Rousseau et al. (96) demonstrated that neutrophils are involved in the early phase of VL by controlling the parasite growth in spleen but not in liver. The authors also found that these cells appear to have no significant effect in late infection in either of these organs. Finally, Almeida et al. (97) found an association between the severity of canine VL, superoxide production and neutrophil apoptosis, observing that neutrophil function alters according to disease stage. Initially, there is a high level of ROS production, ensuring resistance to infection. However, in the late phase of the disease, ROS production overcomes the antioxidant capacity of the cell, leading to oxidative stress followed by neutrophil apoptosis. The increase in apoptosis and the consequent decrease in oxidative activity results in a predisposition to coinfections, commonly seen in severe cases of canine VL (98). In support of this hypothesis, treatments based on antioxidants have generated good results in patients with leishmaniasis (98). In short, like in CL, neutrophils are part of the complex web that governs the establishment and development of VL in different hosts.

Further studies are needed to clarify the role of neutrophils in the control or proliferation of different species of *Leishmania*. Because the infection phase is crucial, time-dependent investigations of ROS production should be carried out, and the regulatory/inflammatory profiles of Th17 cell participation should be explored in different hosts, considering tissue peculiarities.

## Th17 and Neutrophils can Contribute to Parasite Clearance or Disease

Recent studies have advanced our knowledge about the influences of each parasite/host factor in the pathogenesis of human leishmaniasis. The characterization of the CD4^+^ T helper cell population Th17 has further added to the complexity of host–pathogen interactions, previously thought to be determined by Th1 or Th2 polarization. Currently, participation of innate immune responses and complicated interactions between cytokines secreted by different T cell profiles, as well as the diversity of *Leishmania* species and hosts, are known to be relevant to disease outcome.

Neutrophils can potently protect against or enhance infections and can likewise behave as Trojan horses, supporting the invasion of macrophages by *Leishmania*. In addition to DCs and other components of the innate defense system, neutrophils also regulate T cell polarization, through cytokine secretion, thus establishing a link between innate and adaptive immunity during parasitic infection. Although neutrophil presence has been mostly associated with parasite depletion and cure in *L. donovani* and *L. infantum* infections, the infiltration of these cells is suggested to cause more severe disease presentations in tegumentary forms of leishmaniasis. The reviewed data show that regulatory cytokines such as IL-10 and IL-4 are needed to promote the equilibrium in ROS production, which is enhanced by the presence of neutrophils in chronic infections. In the liver and spleen, *L. infantum/donovani* elicits anti-inflammatory cytokines and stimulates arginase production, leading to control of inflammation while neutrophils and activate macrophages kill parasites. The data also suggest that neutrophil participation depends on the timing of their recruitment, the tissue infected, and their duration at the infection site.

Current understanding of the role of Th17 cells in the progression of pathogenesis or contribution to host-protective immunity is evolving rapidly. We now know that *Leishmania* infections trigger CD4^+^/IL-17^+^ T cell differentiation in mice and humans. The IL-17 function in neutrophil recruitment has been widely demonstrated in experimental models of leishmaniasis, in which strong inflammatory infiltrates are observed. Although mice are more dependent than humans on IL-17 to combat *L. infantum*/*L. donovani* infections, IL-17 has also been associated with improved recovery from VL in humans. Conversely, higher levels of IL-17 during commitment in skin in patients with PKDL, CL, and ML than in healthy controls suggest that in this tissue, IL-17 contributes less to healing than in other tissues. Most of the current data link the IL-17–neutrophil axis to disease development or lesion exacerbation in CL. Nevertheless, differences in immunomodulation between parasite species, and contradictory results in humans, point to IL-17 stimulatory (IL-23) or regulatory (e.g., IL-27, IL-10, and IL-13) cytokines, which may be important in determining how long IL-17 remains present. Similarly to the role of IL-12 in the context of Th1 generation, identifying the role of these cytokines in the development or maintenance of Th17 is essential for understanding the influence of its persistence at lesion sites.

## Author Contributions

SG-d-A and MP-C concept and drafted the paper. SG-d-A, RS, and LT-S acquired, interpreted data, and wrote the paper. SG-d-A, RS, VL, RM, and MP-C revised critically for intellectual content. All the authors approved the final version to be published. All authors agreed to be accountable for all aspects of the work in ensuring that questions related to the accuracy or integrity of any part of the work are appropriately investigated and resolved.

## Conflict of Interest Statement

The authors declare that the research was conducted in the absence of any commercial or financial relationships that could be construed as a potential conflict of interest.

## Footnotes

^1^IL-17 is the founding member of the IL-17 family of cytokines, which comprises IL-17A (commonly known as IL-17), IL-17B, IL-17C, IL-17D, IL-17E (also called IL-25), and IL-17F (45). The most widely investigated and understood cytokines of this family are IL-17A and IL-17F, which play an essential role in host defenses against microbial infections and are implicated in various inflammatory conditions such as autoimmune diseases, metabolic disorders, and cancer (46).
